# Offering an auto-play feature likely increases total gambling activity at online slot-machines: preliminary evidence from an interrupted time series experiment at a real-life online casino

**DOI:** 10.3389/fpsyt.2024.1340104

**Published:** 2024-02-02

**Authors:** Jakob Jonsson, Per Carlbring, Philip Lindner

**Affiliations:** ^1^ Centre for Psychiatry Research, Department of Clinical Neuroscience, Karolinska Institutet, & Stockholm Health Care Services, Region Stockholm, Stockholm, Sweden; ^2^ Department of Psychology, Stockholm University, Stockholm, Sweden

**Keywords:** auto-play, online casino, gambling, policy, time series

## Abstract

Auto-play is a ubiquitous feature in online casino gambling and virtual slot machines especially, allowing gamblers to initiate spin sequences of pre-set length and value. While theoretical accounts diverge on the hypothesized causal effect on gambling behavior of using the auto-play feature, observational findings show that this feature is used to a higher degree by problem and/or high-intensity gamblers, suggesting that banning this feature may constitute a global responsible gambling measure. Direct, experimental research on causal effects of offering auto-play at online casinos is however lacking. Here, we report the findings of an interrupted time series experiment, conducted at a real-life online casino in Sweden, in which the auto-play feature was made available during a pre-set duration on 40 online slot machines, with 40 matched slots serving as control. Aggregated time series on daily betted amount, spins and net losses were analyzed using a structural Bayesian framework that compared observed developments during the peri-intervention period to modeled counterfactual estimates. Results suggest that offering an auto-play feature on online casinos likely increases total gambling activity in terms of betted amount (approx.+ 7-9%) and (perhaps) number of spins (approx. +3%) but has no effect on net losses. Limitations of studying auto-play effects on a population-level, as well as the complexities of banning this feature within a complex ecosystem of non-perfect channelization to licensed providers, are discussed, including suggestions for future research.

## 1 Introduction

Commercial gambling has undergone significant changes in the last 20 years, most prominently a widespread transition from on-site to digital gambling. However, types and popularity of games have remained the same, only digitalized, and so has the associations between specific gambling types and risks of developing and maintaining gambling problems. Today, online gambling is by far the most prevalent type among Swedish treatment-seekers, prominent among which is online casino games (1). Structural characteristics such as game speed, repeatability, availability and phenomenon like losses disguised as wins (2, 3), are well-recognized to increases risks of developing problem gambling (4–6).

Moreover, the format and user experience of digital casino games make it easy to incorporate secondary features, which may or may not be as locally regulated as for example randomness mechanisms, return to player rates and similar core game characteristics. One common such secondary feature is auto-play, which offers the gambler the opportunity to commit to a fixed number of rounds that are then executed sequentially and automatically on behalf of the player, if not actively aborted (7). Notably, theoretical accounts diverge on whether this feature can be expected to increase or decrease (problematic) gambling behaviors. On one hand, auto-play may increase the risk for dissociation while gambling, promote prolonged and faster gambling, give the illusion of less control (auto-play sequences can typically be aborted at any time), and enable and parallel gambling (7), thereby increasing gambling activity. Since autoplay does not alter the underlying randomness mechanism or return to player rate (or volatility), increased gambling activity should (on average) equate to more money spent, thereby increasing the risk and magnitude of negative consequences. On the other hand, from a behavioral analytic perspective (5), auto-play seemingly softens the response-outcome contingency by introducing a greater temporal delay between behavior (sequence onset) and the consequences thereof, while also lowering the overall frequency of the behavior (response). This may theoretically decrease the potential for learning and maintaining this particular behavior. Moreover, the gambling industry has argued that the auto-play feature could be seen as a pre-commitment tool of sorts, that they argue increases player control (UK 8).

Of importance, these factors are not mutually exclusive – at least not over time for individual gamblers, or on a population-level – and the overall impact of making auto-play available thus needs to be examined empirically, both on an individual and population level. Past research has shown that problem and at-risk gamblers show higher use of auto-play than non-problem gamblers, but after controlling for other factors, auto-play did not predict gambling problems (9). Surveys with help-seeking populations indicate a high use of auto-play, and that it was perceived to have a role in over-consumption and loss of control (UK 8). In a qualitative study of arcade slots gamblers, Husain et al. (10) found differences in use of auto-play such that high-control gamblers tended not to use it, while low-control gamblers used it when getting tired during long gambling sessions and believed (erroneously) that auto-play would increase the probability of winning.

In a public health initiative to combat excessive gambling, the UK Gambling commission has recently introduced a ban on auto-play features on online slots operating on the domestic market, stating that “The structural use of auto-play and its potential to facilitate play on multiple products at once does not seem compatible with the requirements for operators to keep players safe” (8, p. 21). After analyzing real-world gambling data, the commission reported that auto-play stakes tend to be on lower on average than total average stakes, apparently driven by a higher prevalence of smaller stakes using auto-play. Moreover, there was a negative correlation between stake size and use of auto-play. Sessions that included auto-play were longer in comparison to total session averages, yet there was a negative correlation between risk scores and proportion of auto-play, and a non-linear relationship between auto-play usage and financial loss: financial loss decreased with to 30% use of auto-play but increased past a proportion of 40% auto-play spins and above (8). In 2022, the Swedish Gambling Authorities (11) proposed a similar ban on auto-play features. Recent survey data from Sweden suggests that auto-play is used by over 80% of daily gamblers (who can be expected to have a greater prevalence of problem gambling), almost twice that of weekly- or monthly gamblers (12).

In sum, the extant theoretical and empirical literature suggests a complex association between the use of auto-play features and the presentation and prevalence of problem gambling. Of note, there is a striking lack of research on the causal effect of offering auto-play per se: past research is arguably insufficient in explaining whether auto-play actively promotes problematic gambling behaviors (e.g. betting more than they would otherwise do) or is merely used as a strategy by some gamblers who are already at-risk. The former would suggest that a universal ban of auto-play would constitute a responsible gambling measure, while the latter would suggest that only identified at-risk gamblers would need to be covered by a ban. Conversely, should auto-play be shown to have no effect on gambling, or even have a protective effect (as often argued by the gambling industry), a ban could be considered an unnecessary restriction which may risk hurting channelization to licensed venues (notwithstanding research showing that non-problem gamblers are typically not disturbed by responsible gambling measures; 13).

Evidence-based public health policy requires applicable and generalizable real-world findings to appropriately balance different interests. In the current study, we describe the results of an interrupted (structured) time series experiment, conducted at a real-life online casino, with the aim of estimating the population-level causal impact of offering an auto-play feature on slot machines on immediate gambling behaviors and outcomes.

## 2 Methods

### 2.1 Setting, design, and ethics

The experiment was an initiative from ATG – a large, licensed gambling provider in Sweden – and was conducted as a live A/B test within the legal framework of service development to comply with responsible gambling regulations as required by the Swedish Gambling Act (SFS 2018:1138). While historically and still predominantly a provider of horse betting (which in 2022 accounted for 79% of net revenue), ATG also has a license to offer sports betting (12% of 2022 net revenue) and casino games (10% of 2022 net revenue). At time of the experiment, ATG was one of few on the Swedish market that had not already implemented the auto-play feature; thus, the experiment did not involve exposure to any gambling element not already theoretically available to all Swedish customers. Indeed, research suggests that gambling on multiple platforms is a risk factor for showing problem gambling (14). The A/B test was approved after review by the ATG-internal Responsible Gambling Board and permission to share (aggregated) data from this A/B test for research purposes was granted by the Swedish Ethical Review Authority (dnr 2020-01870 and 2021-03657). Transfer and use of aggregated data (i.e. no personal data) was further regulated by a signed academia-industry collaboration agreement that guaranteed full academic freedom.

The experiment was initially conceived as a traditional A/B test with randomized allocation of individual gamblers; however, this study design was later abandoned for the following reasons: (1) the A/B testing tool did not allow allocation at the account-level, only at the device-level, entailing that gamblers using multiple devices could receive both allocations during the study period, confounding account-level statistics; (2) during planning, many popular web browsers began automatically deleting cookies, cache and other temporary files, further risking consistent allocation of individuals; (3) it was not possible to log individual spins as being either manual or automatic, entailing that causal effects of actually using the auto-play feature, compared to merely having it available (akin to the intention-to-treat principle in clinical trial), would not be possible to estimate in any case; and (4) sharing aggregated as opposed to individual account data was deemed preferable from an integrity perspective.

Instead, a Bayesian structural time series design was adopted wherein collections of slot machines either gained the auto-play feature (intervention arm) or not (control arm) during a pre-set period, before and after which this feature was not available at any slot machine. I.e., allocation was at the level of slot machine, not individual gamblers. The favored Bayesian structural time series design, popular in public health research (e.g. 15, 16), allows causal inferences about the impact of introducing an intervention by analyzing the difference between the expected (i.e. counterfactual) data and the observed data during the intervention period, the former calculated by using both the pre-period time series as well as the continued time series of the control arm (17). See below for details on analyses. Pandemic-related temporary gambling legislation was in effect in Sweden during the pre- and peri-intervention period, which included a mandatory deposit limit (per gambling provider) of 5000 SEK per week, roughly corresponding to a Swedish net median income. The post-intervention period was synchronized with lifting of the temporary legislation, for ethical reasons.

### 2.2 Experimental arms

**Figure 1:**
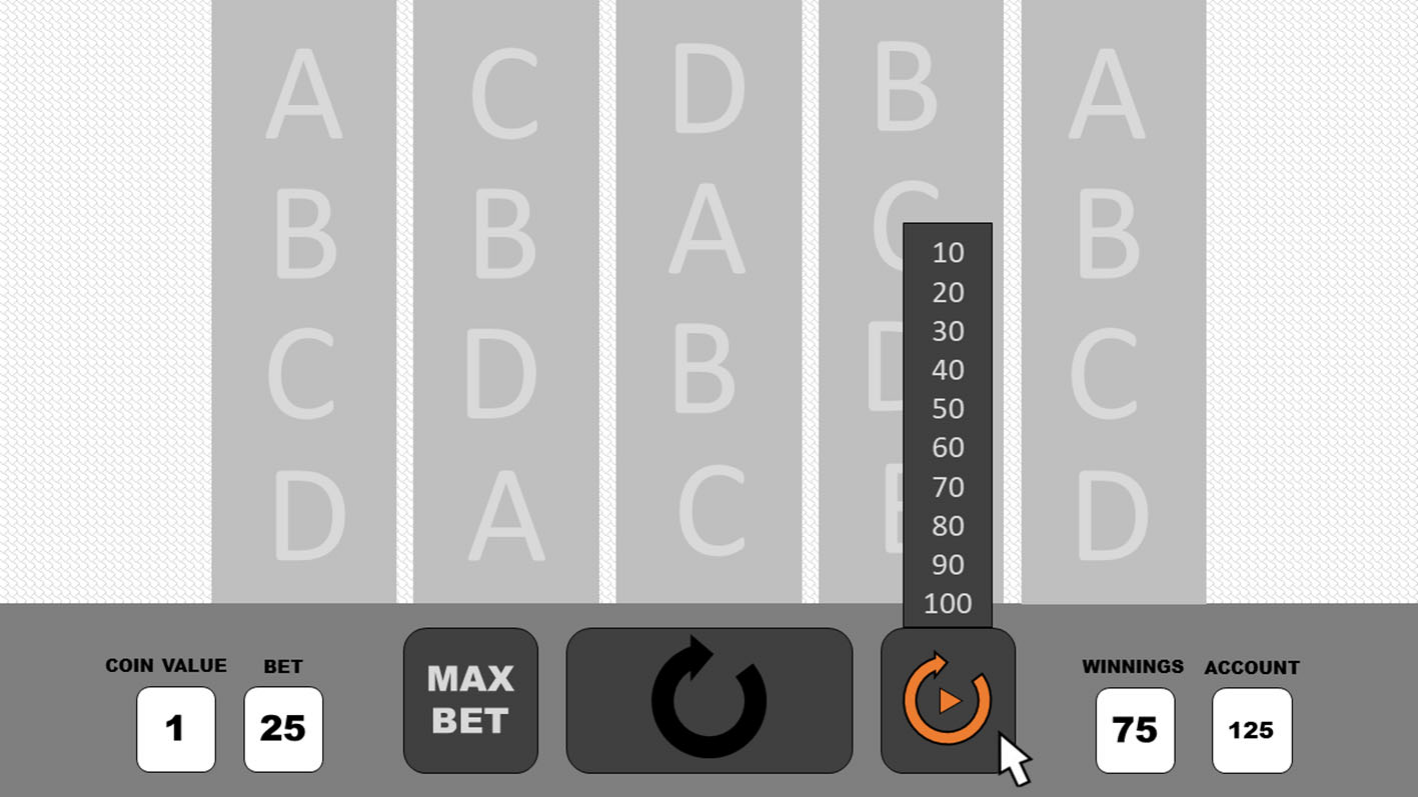
Schematic mock-up of how the auto-play feature was presented in one game. Schematic mock-up provided instead of screenshots for copyright reasons. Different slot machines have different GUI and therefore different feature presentation. Some slot machines had other options for setting number of auto-play turns. Components included in above mock-up (not exclusive) are not exact to scale.

Both from a user experience and technical perspective, the auto-play feature in essence replaced button presses: an initiated spin sequence could be aborted at any time and did not entail a hard monetary commitment at initiation. Initiated spin sequences continued with a delay of three seconds in-between spins (including spin and win animations), as per Swedish legislation. Although specific layout and features varied somewhat between auto-play enabled games, most offered a pre-set option of 10—100 auto-initiated spins (in increments of 10), with the button placed in near vicinity of the (manual) spin button. See **Figure 1** for a schematic mock-up showcasing how this was presented in one game.

### 2.3 Data and statistical analyses

**Figure 2:**
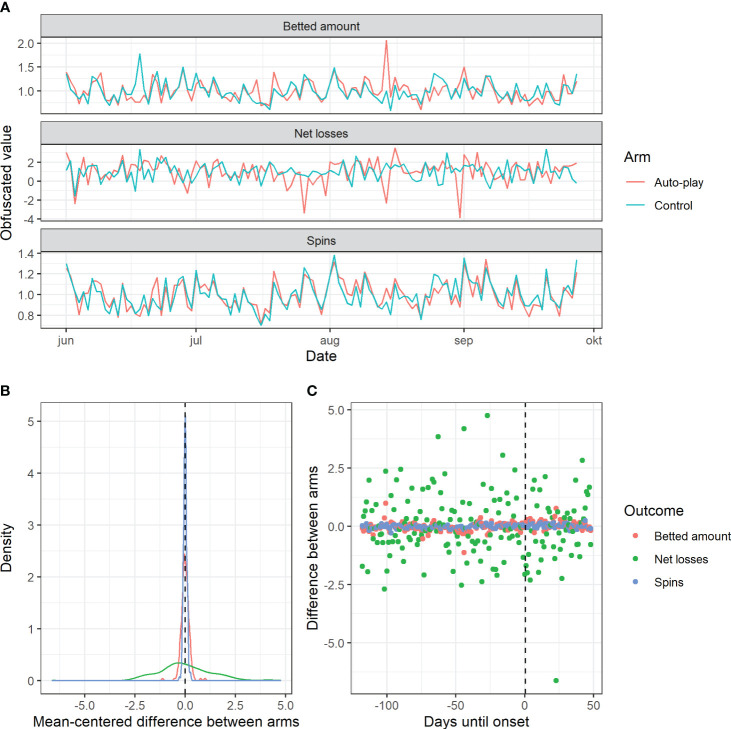
Equivalence of intervention and control time series prior to introducing intervention. Plotted values mean-standardized within-arms for visualization and preprocessing purposes only; for analyses, package-default standardization was used, equivalent to the Bayes approach to setting priors. **(A)** Raw obfuscated values (per outcome) over time. **(B)** Density plots of differences per arms (per outcome). **(C)** Same difference scores plotted against time until onset.

Prior to data collection, the gambling provider identified 40 + 40 slot machines from two separate game providers that showed similar trends prior to data collection, see **Figure 2**. Stratifying by developer was necessary since the auto-play feature was introduced as an option by one of these, thereby enabling the experiment. Data were then collapsed by summing across outcome and arm, creating 3 × 2 time series covering a period of 167 days (units): betted amount, net losses, and number of spins. Since it is common among gamblers at ATG to play casino games while also engaged in betting, only outcomes specific to casino gambling were included, excluding other outcomes, common in the research field, from consideration (e.g. deposit derivates). To allow public release of proprietary information, all-time series were obfuscated through conversion to pseudo-currency and pseudo-counts by random number division, prior to data sharing.

Analyses were run in the R (4.1.1) statistical environment, using the CausalImpact R-package (17) to estimate the effect of introducing auto-play by comparing observed values during the peri-period from the counterfactual estimated from the pre-period time series as well as peri-period developments in the control arm. Due to complexities in estimating nested seasonality, we opted to run separate models that included either weekly or monthly seasonality: the former since gambling activity is typically higher on weekends, and the latter since gambling activity is often higher around the 25^th^ of each month (a common payday in Sweden), reflected in observed greater gambling activity then (18). Number of MCMC samples to draw was set to 10 000, and static regression was used to protect against overspecification.

Although the study design in theory allows examining also the return to normal (peri to post), we opted not to include such analyses for the following reasons: (i) for ethical reasons, the removal of the auto-play feature was designed to coincide with the lifting of the temporary COVID-19 responsible gambling legislation in Sweden (which had included a hard deposit limit), likely resulting in large-scale changes in gambling habits that would confound estimates of causal effects of removing the auto-play feature specifically; (ii) use of the control time series covering the peri-post period would arguably be inappropriate since it would require an assumption of no carry-over effects after the intervention, which in itself is a research question; (iii) the intervention period was deemed too short to be the sole source of estimates of seasonality and general trend.

## 3 Results

Plotting time series of the two arms revealed similar periodicity and within-arm variance, see panel A of **Figure 2**. Density plots of the between-arm differences in mean-centered values across arms revealed symmetrical distributions (panel B1 of **Figure 2**) and plotting these difference values against time-to-onset revealed no obvious pattern (panel B2 of **Figure 2**), consistent with choice of onset-date being quasi-random.

Bayesian structured time series analyses revealed that regardless of seasonality adjusted for, or priors used, there was a significant 6.9—9.1% increase (depending on model) in betted amount after introducing the auto-play feature. Of note, in the robust priors-model with weekly seasonality, the Bayesian p-value was p<.05, but the credibility interval marginally covered zero; such cases are theoretically possible whenever probabilities are empirically estimated using random draws, and the distribution is not perfectly symmetrical. *Post-hoc* exploratory analyses – consistent with the Bayesian, as opposed to frequentist framework – revealed that the lower bound of the credibility interval crossed zero when using priors of 0.015.

Regarding net losses, no significant effect was revealed, regardless of model. A seasonality-robust, roughly 3% increase in number of spins was detected when using standard priors, but this effect was not significant when using robust priors. See **Table 1** for full results.

**Table 1:** Estimated relative effects of introducing auto-play.

	Relative effect (95% CI) and Bayesian posterior tail probability			
	*Weekly seasonality included*		*Monthly seasonality included*	
	Standard prior	Robust prior	Standard prior	Robust prior
Betted amount	+6.9% (0.62—14%), p_Bayes_=.0149	+6.9% (-0.88—16%), p_Bayes_=.0424†	+8.7% (2.2—16%), p_Bayes_=.0044	+9.1% (0.92—18%), p_Bayes_=.0146
Net losses	-8.9% (-30—22%), p_Bayes_=.2199	-8% (-33—33%), p_Bayes_=.2627	-8.6% (-29—23%), p_Bayes_=.2211	-7.9% (-33—34%), p_Bayes_=.261
Spins	+3.2% (0.15—6.4%), p_Bayes_=.0202	+1.9% (-2.1—6.2%), p_Bayes_=.1762	+3.4% (0.34—6.6%), p_Bayes_=.01547	+2.2% (-1.8—6.5%), p_Bayes_=.14795

^†^Note that probability estimates and credibility intervals may not always agree when probabilities are calculated from randomly drawn, asymmetric distributions. Standard priors were package-default 0.01, while robust were 0.02.

## 4 Discussion

The current study provides preliminary experimental evidence that offering an auto-play feature on online casinos does indeed increase total gambling activity in terms of bets and (perhaps) spins on a population-level, but not net losses. The latter null result is not unexpected since net losses are partially determined by winnings (return to player rates of slot machines are typically around 95%), which increases random variation and hence statistical noise. Overall, these findings are congruent with previous observational research and – assuming applicability of the total consumption model of gambling – are also congruent with both recent (8) and suggested legislation (11) in different jurisdictions that bans this feature on online casinos, in public health initiatives to prevent excessive gambling.

However, this first study was performed on population-aggregated data and thus cannot by design provide insights into the mechanisms behind the observed effect on an individual level, i.e. what type of gambling (e.g. fewer larger bets versus smaller but more frequent bets), by what type of gambler (e.g. high vs low-intensity), that drove the population-level change. Based on previous research showing that problem and/or high-intensity gamblers are more likely than recreational gamblers to make use of the auto-play feature (8, 9, 12), we can hypothesize that the apparent increase was driven, presumably to a considerable degree, by increased gambling by problem gamblers. This however remains to be shown empirically in studies featuring account-tracking and random allocation at the account-level. With individual-level data, split by auto-play use, not only could outcomes be contrasted between arms, but it would also be possible to estimate the direct causal effect of actually using the feature using e.g. the Complier Average Causal Effects framework (19). This is particularly important, since not all players will use the autoplay feature even if available. Of note, these analyses would require objective log data on whether auto-play was used or not, at least on a session-level but preferably bet-level, to protect against misclassification bias due to within-session variation. Such data was unfortunately not available in the current study (see above for extended rationale for study design) but should be considered a priority for future research. With individual account data, it would also be possible to calculate a better estimate of the additional money lost due to auto-play usage, since population-level daily aggregates of net losses are primarily driven by chance outcomes that day – in the current study also contingent on daily within-arm popularity of different slot machines – rather than specific gambling behaviors.

While findings of the current study suggest that removing the auto-play option may constitute an effective preventive measure (applied globally or targeted towards flagged at-risk gamblers), it is important to note that current study was not designed to answer the greater question of how such a ban would work in a complex, multi-actor system with non-perfect channelization to legal gambling avenues – this includes Sweden, the context in which the current study was performed. Prior research shows that auto-play is used to a greater extent by problem gamblers (9), who in turn are those most likely to respond negatively to responsible gambling measures (13). However, to our knowledge, there has been no prior research on how an auto-play ban specifically would be perceived by different groups of gamblers. Of note, recent Swedish survey data suggests that even among gamblers who gamble only a few times a month or quarterly, self-reported use of autoplay ranged from roughly 20-40-% (12). Since the same survey data reports that only around 10% of gamblers can tell whether a gambling operator is licensed or not, it cannot be ruled out that an auto-play ban on legal gambling platforms will temporarily decrease channelization by driving gamblers (both recreational and problem) to non-licensed platforms that still offer auto-play and may not have any duty of care obligations at all. However, even if so (which remains to be estimated empirically), this risk would need to be balanced against a likely long-term benefit of not allowing new gamblers to ever become acquainted with this feature. In lieu of multi-source account data covering both licensed and unlicensed providers (e.g. from online payment gateways, 20), one feasible way to estimate the outcome of this balancing act would be to survey attitudes among representative gambling customers, combined with statistical estimation of the actual causal effects (19) of offering auto-play.

In sum, findings from the current study suggests that offering auto-play likely does increase overall gambling activity on online casinos, yet more research – using other study designs – is needed to elucidate the mechanisms behind this population-level effect.

## Data availability statement

The original contributions presented in the study are included in the article/supplementary material. Further inquiries can be directed to the corresponding author.

## Ethics statement

The studies involving humans were approved by Swedish Ethical Review Authority. The studies were conducted in accordance with the local legislation and institutional requirements. The ethics committee/institutional review board waived the requirement of written informed consent for participation from the participants or the participants’ legal guardians/next of kin because data was collected within the framework of service development at the gambling provider; ethical approval was granted for sharing aggregated data for research purposes.

## Author contributions

JJ: Conceptualization, Funding acquisition, Investigation, Methodology, Writing – review & editing. PC: Conceptualization, Methodology, Writing – review & editing. PL: Conceptualization, Data curation, Formal analysis, Funding acquisition, Investigation, Methodology, Project administration, Resources, Software, Supervision, Validation, Visualization, Writing – original draft, Writing – review & editing.
